# Hydrogen-Bonding Interactions in T-2 Toxin Studied Using Solution and Solid-State NMR

**DOI:** 10.3390/toxins3101310

**Published:** 2011-10-21

**Authors:** Praveen Chaudhary, Roxanne A. Shank, Tony Montina, James T. Goettel, Nora A. Foroud, Paul Hazendonk, François Eudes

**Affiliations:** 1 Department of Chemistry and Biochemistry, University of Lethbridge, 4401 University Drive West, Lethbridge AB T1K 3M4, Canada; Email: praveen.chaudhary@uleth.ca (P.C.); shankr2@uleth.ca (R.A.S.); tony.montina@uleth.ca (T.M.); james.goettel@uleth.ca (J.T.G.); 2 Lethbridge Research Centre, Agriculture and Agri-Food Canada, 5403-1st Avenue South, Lethbridge AB T1J 4B1, Canada; Email: nora.foroud@agr.gc.ca

**Keywords:** T-2 toxin, trichothecene, NMR, hydrogen-bonding, ribosome, toxin, epoxide, water bridging, deuterium exchange, chemical exchange

## Abstract

The structure of T-2 toxin in the solid-state is limited to X-ray crystallographic studies, which lack sufficient resolution to provide direct evidence for hydrogen-bonding interactions. Furthermore, its solution-structure, despite extensive Nuclear Magnetic Resonance (NMR) studies, has provided little insight into its hydrogen-bonding behavior, thus far. Hydrogen-bonding interactions are often an important part of biological activity. In order to study these interactions, the structure of T-2 toxin was compared in both the solution- and solid-state using NMR Spectroscopy. It was determined that the solution- and solid-state structure differ dramatically, as indicated by differences in their carbon chemical shifts, these observations are further supported by solution proton spectral parameters and exchange behavior. The slow chemical exchange process and cross-relaxation dynamics with water observed between the hydroxyl hydrogen on C-3 and water supports the existence of a preferential hydrogen bonding interaction on the opposite side of the molecule from the epoxide ring, which is known to be essential for trichothecene toxicity. This result implies that these hydrogen-bonding interactions could play an important role in the biological function of T-2 toxin and posits towards a possible interaction for the trichothecene class of toxins and the ribosome. These findings clearly illustrate the importance of utilizing solid-state NMR for the study of biological compounds, and suggest that a more detailed study of this whole class of toxins, namely trichothecenes, should be pursued using this methodology.

## 1. Introduction

Trichothecenes are a group of toxic sesquiterpenoidal fungal metabolites composed of a central core of fused cyclohexene/tetrahydropyran rings, with a cyclopentyl moiety fused to the tetrahydropyran ring through C-2 and C-5, and an epoxide function at C-12 [1]. Over 200 trichothecenes have been identified primarily from fungi [2], and can be classified into one of four groups (types A, B, C and D) based on structural features. Type A (T-2 and HT-2 toxins) and type B (nivalenol (NIV) and 4-deoxynivalenol (DON)) trichothecenes are differentiated from one another by the absence or presence of a ketone at C-8, respectively [1]. Type A and B trichothecenes are produced by phytopathogenic *Fusarium* spp. that are capable of causing diseases of important food crops, including Fusarium Head Blight (FHB) and root rot of cereals and maize, which can lead to significant yield losses in infected crops [3]. The trichothecenes are synthesized by the fungus during the course of infection and can accumulate in developing kernels [1]. Type C trichothecenes (crotocins), are the least toxic group and are characterized by a second epoxide functionality at C-7/C-8 or C-9/C-10 [4]. Type D trichothecenes (satratoxins and verracurins), are synthesized primarily by *Stachybotrytis* spp., and possess an ester-linked macrocycle at C-4 to C-15 and are the most toxic among trichothecenes [4,5]. Considerably less attention has been given to the types C and D trichothecenes, since they are not associated with food-crop pathogens and are subsequently less likely to pose a threat to the human public. 

Type A and B trichothecenes have been found to contaminate foodstuffs [6,7] and are known to cause a potentially lethal condition in humans called alimentary toxic aleukia (ATA) characterized by early stages of severe gastrointestinal irritation followed by a marked reduction in leukocyte counts and subcutaneous hemorrhaging [8,9]. At the cellular level, trichothecene exposure can lead to inhibition of protein [10,11], RNA, and DNA synthesis [12], disruption of membrane integrity [13,14], cell division [15], and mitochondrial function [16,17], and can induce apoptosis in mammalian cells [17,18]. However, the mechanism for toxicity of these interactions is not well understood.

**Figure 1:**
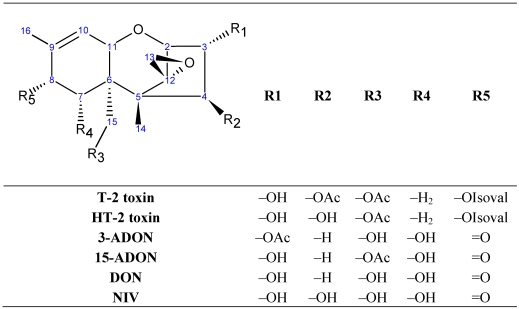
Structures of type A and B trichothecenes. Type A trichothecenes include T-2 toxin, and HT-2 toxin. Type B trichothecenes include nivalenol (NIV), 4-deoxynivalenol (DON), 3-*O*-acetyl DON (3-ADON), 15-*O*-acetyl DON (15-ADON). OAc = acetyl function; OIsoval = isovalerate function.

The best studied interaction of trichothecene toxicity thus far, has been the inhibition of protein synthesis. Trichothecenes are known to restrict 60S ribosome function through an interaction with the ribosomal protein L3 (RPL3) [19]. RPL3, a mostly globular protein, has an extended domain referred to as a “W finger” that makes contacts with the A- and P-loops of the peptidyl transferase center (PTC), and functions along with an *N*-terminal extension, which contacts the opposite side of the A-site, and is believed to coordinate ribosomal function [19]. Meskaukas and Dinman recently proposed a model for how the three domains of RPL3 function together as a “rocker switch” to coordinate amino acyl-tRNA (aa-tRNA) and 25S rRNA in a stepwise process during translation elongation [20]. Therefore, an interaction of trichothecene toxins with the W-finger or *N*-terminal extension of RPL3 would result in an inhibition of protein synthesis in eukaryotic cells [4,21]. Some trichothecenes can inhibit the initiation step of protein synthesis (type I inhibitors), while others inhibit the elongation (type E) or termination step (type T) [4]. Structural features of the trichothecenes can determine the mechanism of inhibition of protein synthesis; for example, the presence of an oxygen functionality at C-15 favors type I inhibition. T-2 toxin and nivalenol (NIV), both type I inhibitors, have an acetyl and a hydroxyl function at C-15, respectively [22,23]. Type E and T inhibitors are differentiated by the presence or absence of a hydroxyl substituent at C-4, respectively [24]. The trichothecene core structure is depicted in Figure 1, along with the substituent groups of key A and B type trichothecenes.

DON is the most prevalent toxin associated with FHB and is subsequently the most prevalent trichothecene found to accumulate in grain products [1]. Screening for DON contamination in food-stuffs is standard practice in developed countries. On the other hand, there are no regulations in place for T-2 toxin screening. While detection of T-2 toxin in grain products is not as commonly observed as DON, in recent years, increasing reports of T-2 and HT-2 toxin contamination in grain crops throughout Europe have been published [7,25,26,27]. This increased observation of T-2 and HT-2 toxin is particularly widespread in oat crops, which may be more susceptible to type A trichothecene producers such as *F. sporotrichioides* and *F. langsethia* [28]. The increased observation of T-2 toxin is of great concern since this type of trichothecene is roughly ten times more toxic in mammalian systems than DON [29]. Therefore, acquisition of a more detailed understanding of the toxicity of trichothecenes at the molecular level may ultimately lead to the development of trichothecene resistant crops. 

In order to better understand the mechanisms of inhibition and cytotoxicity of these compounds, detailed insights into the hydrogen bonding behavior of this class of molecule, both in the solution- and solid-state, are required. X-ray crystallography indicates that there may be some weak intermolecular hydrogen bonding occurring between the hydroxyl oxygen at C-3 and the oxygen in the tetrahydropyran ring. However, due to the low resolution of the structure, no direct evidence for the hydrogen bonding interaction is available. Solution NMR structures provided thus far, have focused primarily on spectral identification rather than determining three-dimensional configuration and interactions [30]. It is therefore imperative to obtain a complete assignment of the T-2 toxin ^1^H and ^13^C spectra in solution-state and make direct comparisons with solid-state ^13^C NMR data to look for differences in their structure and in their inter- and intra-molecular interactions.

## 2. Materials and Methods

### 2.1. Solution-State NMR Measurements

All spectra were acquired at ambient temperature (25 °C) using a Bruker Avance 300 spectrometer operating at a magnetic field strength of 7.05 Tesla, giving rise to a Larmor frequency of 300.131 MHz for ^1^H and 75.468 MHz for ^13^C nuclei, outfitted with a 5 mm HX PABBO BB probe. T-2 toxin (SIMGA, EC 244-297-7) was dissolved in deuterated chloroform (CDCl_3_) to a concentration of 1 mg/mL using tetramethylsilane (TMS) as an internal reference for both ^13^C and ^1^H nuclei. It has been demonstrated by other groups [31,32,33] that trichothecene conformation is highly dependent on solvent; thus, it is important to note here that the CDCl_3_ was dried over sodium sulfate in order to prevent water contamination. The 1D ^1^H spectrum was recorded with a 90° pulse width of 12.4 μs, a recycle delay of 1.0 s, and 128 transients. The 1D ^13^C spectrum was recorded with a 90° pulse width of 7.6 μs, a recycle delay of 2.0 s, and 8464 transients.

The 2D homonuclear magnitude gradient ^1^H COSY spectrum was acquired in 256 increments covering a spectral width of 1800 Hz (6.0 ppm) in both dimensions, using a recycle delay of 1.5 s. Four transients were collected for each increment, having 1024 points.

The gradient ^1^H NOESY spectrum was acquired in 256 increments covering a spectral width of 1802.45 Hz (6.0 ppm) in both dimensions, using a recycle delay of 1.0 s and a mixing time of 1.0 s. Thirty-two transients were collected for each increment, having 1024 points.

The 2D (^1^H-^13^C) HSQC heteronuclear correlation spectrum was acquired in 128 increments, using a recycle delay of 2.0 s, and a spectral width covering 4006.41 Hz (13.34 ppm) in the direct dimension and 12,500 Hz (165.62 ppm) in the indirect dimension. One-hundred-and-fifty-two transients were collected for each increment, having 1024 points.

The 2D (^1^H-^13^C) HMBC spectrum was acquired in 256 increments using a recycle delay of 2.0 s, and a spectral width covering 1951.60 Hz (6.50 ppm) in the direct dimension and 14,268 Hz (190.24 ppm) in the indirect dimension. One-hundred transients were collected for each increment, having 1024 points.

Deuterium exchange experiments were performed where deuterated water, D_2_O (Cambridge Isotope Laboratories; DLM-2259-250), was added dropwise to the NMR tube between consecutive NMR measurements, over the course of 3 h. 1D ^1^H and 2D ^1^H-^1^H COSY experiments were performed for each drop of D_2_O added. 

### 2.2. Solid-State NMR Measurements

All spectra were acquired at ambient temperature (21.5 °C) using a Varian Inova 500 spectrometer operating at a magnetic field strength of 11.7 Tesla, giving rise to a Larmor frequency of 500.13 MHz for ^1^H and 125 MHz for ^13^C nuclei, outfitted with a 2.5 mm four channel HFXY Varian T3 probe. Solid-state NMR samples for T-2 toxin were prepared as received from the Sigma-Aldrich Chemical company (EC 244-297-7), this highly crystalline sample displayed typical needle-like crystals. The microcrystalline powder was loaded into a 2.5 mm zirconium oxide rotor with a sample volume of 11 μL. The 90° pulse was calibrated at 2.0 μs. Magic angle spinning (MAS) was used to mimic isotropic motion in the sample [34,35]. A high MAS rate of 22.0 kHz was used to reduce dipolar coupling of the nuclei [36,37]. A total of 4096 points were acquired over 40.96 ms using a spectral width of 100 kHz. TPPM ^1^H decoupling was performed with a field strength of 120 kHz for all ^13^C spectra [38,39]. The direct polarization (DP) ^13^C spectrum was acquired with a total of 4096 transients and with a recycle delay of 15 s. The cross polarization (CP) ^13^C spectrum was acquired with a total of 14000 transients and with a recycle delay of 5 s. CP was achieved using the adiabatic method with a contact time of 1.5 ms [40]. The DP and CP ^13^C spectra were processed using linear prediction up to 64 k-points.

### 2.3. Spectral Simulations and X-ray Modeling

The 300 MHz ^1^H spectra were simulated using SpinWorks software [41]. The FID’s were zero-filled four-fold and subjected to Gauss-Lorentz apodization with a line broadening of −1.00 Hz, and a Gaussian broadening of 0.1. The spectra were simulated in 4 parts as the whole spin system could not be simulated at once. The isovalerate group, containing the hydrogens H-1', H-2'_A_, H-2'_B_, H-3', H-4' and H-5', was simulated as a nine-spin ABCD_3_E_3_ system. Similarly the six-membered ring, containing hydrogens, H-7_α_, H-7_β_, H-8, H-16, H-10 and H-11, was simulated including the sidechain hydrogens H-15_A_ and H-15_B_ as a ten-spin ABCD_3_EFGH system. The five-membered ring containing the hydrogens, H-2, H-3, H-3_OH_, H-4, H-14, H-13_A_ and H-13_B_, was simulated as a nine-spin ABCDE_3_FG spin system. Long range coupling were considered up to 5 bonds, and inherent line width of 0.3 Hz was used along with Lorentzian line shapes. In the simulation of the isovalerate group 70 transitions were assigned, with an RMS deviation of less than 0.23 Hz and a largest absolute frequency difference less than 0.56 Hz. Standard deviations in all the spectral parameters ranged from 0.05-0.10 Hz. In the simulation of the A ring 2336 transitions were assigned with RMS deviation below 0.034 Hz, with a largest absolute difference of 0.08 Hz. Standard deviations in all the spectral parameters ranged from 0.03-0.06 Hz. In the C ring simulation the 1328 transitions were assigned with an RMS deviation below 0.031 Hz, and largest absolute difference of 0.09 Hz. Standard deviations in all the spectral parameters range from 0.003-0.006 Hz.

## 3. Results

### 3.1. Structural Rigidity and Water Bridging

The central core of trichothecene toxins is composed of several fused rings, which donate a considerable amount of rigidity to the structure; however, the tetrahydropyran, and cyclopentyl rings may still experience a certain amount of flexibility, particularly in solution. Through-bond coupling constants offer a good indication as to the rigidity of a structure.

**Figure 2:**
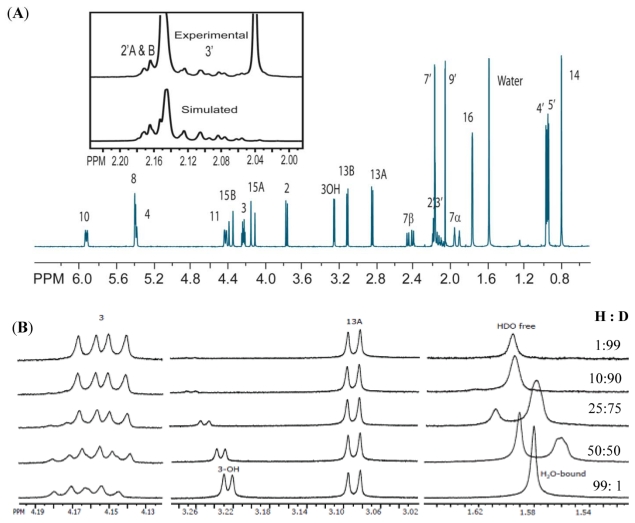
(A) ^1^H NMR solution-state spectrum at 300 MHz in CDCl_3_; (B) Deuterium exchange experiment. Regions exhibiting significant changes throughout the incremental addition of D_2_O have been expanded to show peak structure. Of particular interest, is the H-3 _OH_ and H_2_O/HDO region, which not only demonstrate significant changes in chemical shift, but also exhibit the retention of their sharp peak structure, indicating a slow chemical exchange process. H-3 is less affected in that the only observable changes in the loss of coupling to H-3_OH_ as the latter peak is converted to H-3_OD_.

The solution-state ^1^H spectrum (Figure 2A) was assigned and simulated to accurately determine chemical shifts and coupling constants. The assignment employs the labeling convention by Savard and Blackwell [42]. Coupling constants up to five-bonds were considered, particularly where significant π-electron density was expected. The corresponding chemical shifts and coupling constants are presented in Tables S1-S4. The close agreement between simulated and experimental spectra strongly supports the accuracy of the assignment. The methine (H-3′) and methylene (H-2′) protons of the isovalerate group are strongly coupled, as demonstrated by the highly second order features in the spectrum, shown in the inset of Figure 2A. Despite this complication, it was possible to extract coupling constants and chemical shifts that have not previously been reported.

**Figure 3:**
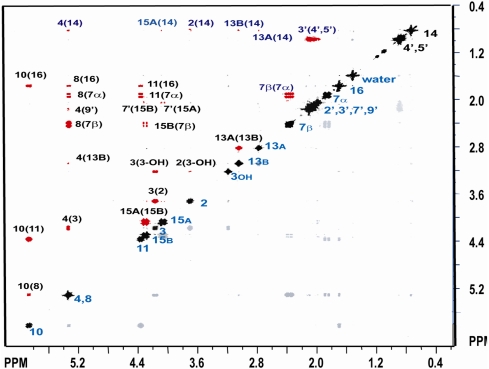
^1^H COSY at 300 MHz in CDCl_3_.

The COSY spectrum (Figure 3) supports the assignment in every aspect and also indicates some long range connectivities of note. The prominent cross peaks in the COSY spectrum (red) represent primarily two- and three-bond connectivities and are listed in Tables S1 and S3. The coupling between H-7_α_ and H-11 strongly supports that the H-7_α_ is in an equatorial position. This four-bond coupling has a large value of -1.59 Hz indicating a W-configuration that is very rigid. A similar coupling between H-7_β_ and H-15_B_ suggest that the H-7_β_ must be in the axial position, and with H-15_B_ pointing towards C-14 and underneath the ring, where the oxygen on C-15 is *gauche* to both C-7 and C-5. This 4-bond coupling is smaller at −0.45 Hz suggesting that there is at least one more rotational isomer of significant probability present in solution, such as one with the oxygen on C-15 being *gauche* to C-7 and *trans* to C-5. In this case one could also appreciate the 4-bond coupling between H-15_B_ and H-11 of −0.59 Hz. 

**Figure 4:**
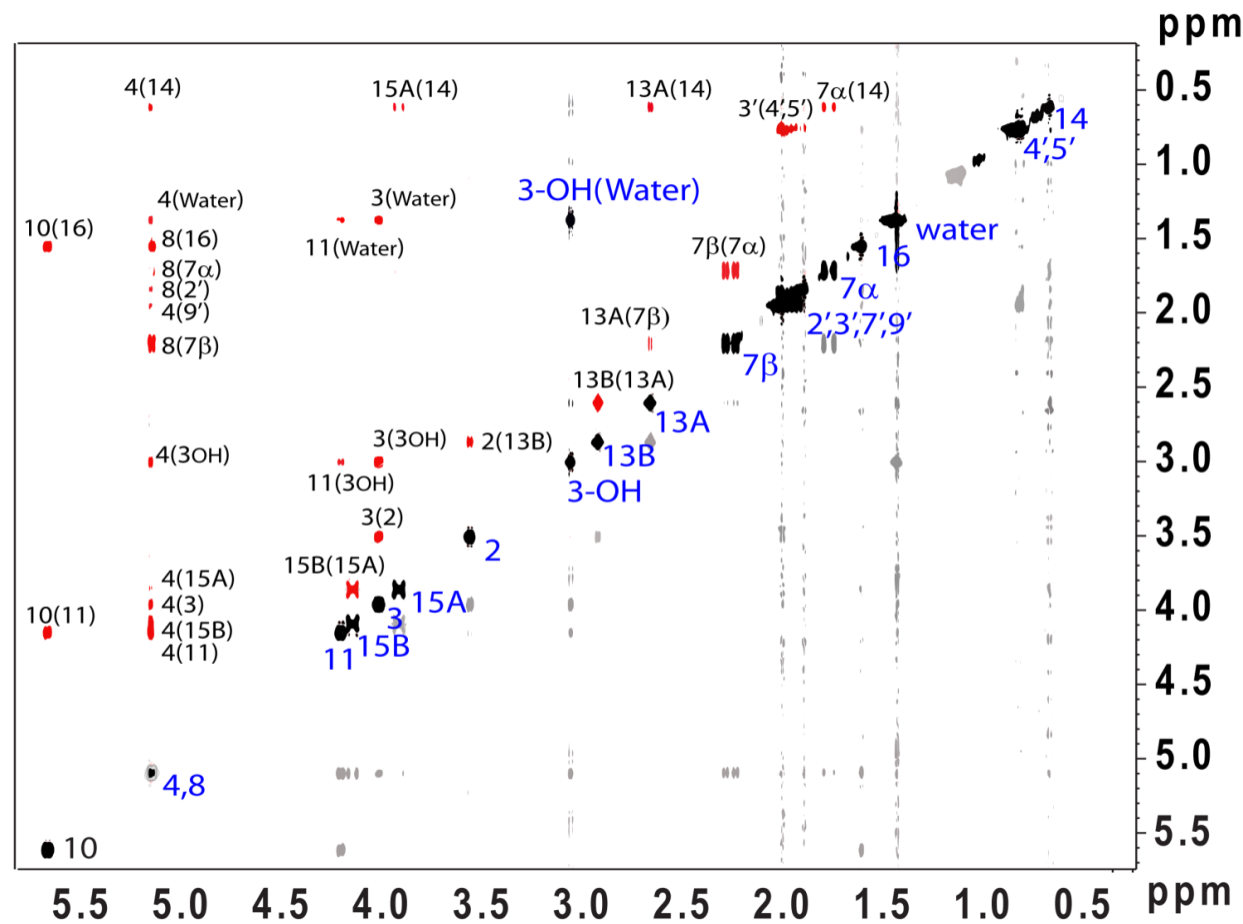
^1^H NOESY at 300 MHz in CDCl_3_. Cross-relaxation peaks bearing a positive phase are shown in red. Negatively phased peaks, including the diagonal autocorrelation peaks and exchange (EXSY) peak observed between H_2_O(H-3_OH_) are shown in black.

The NOESY spectrum (Figure 4) was instrumental in confirming the configuration and in completing the assignment of all the proton resonances, including the methyl resonances of the acetyl side chains, and the specific assignments of the methylene protons (H-7_αβ_, H-13_AB_ and H-15_AB_). Most notably, the cross peaks between hydrogens: H-7_β_ and H-13_A_, H-7_α_ and H-14, H-2 and H-13_B_, H-13_A_ and H-14, H-15_A_ and H-14, H-15_AB_ and H-4, support the assignments made by Greenhalgh *et al.* [43,44,45]. Prominent EXSY crosspeaks were observed between water and H-3_OH_ (Figure 4), indicating the presence of chemical exchange, which was further confirmed with the deuterium exchange experiment (Figure 2B). Furthermore, positive NOESY crosspeaks are observed between the H_2_O peak and H-3, H-4, and H-11. 

A deuterium exchange experiment was performed to determine whether the water is bound to T-2 toxin or whether it is free. Two sharp and rather distinct peaks are observed for H_2_O and HDO (Figure 2B). The linewidth of the H_2_O peak was compared to that of the uncoupled methyl hydrogen on C-14, and is remarkably similar, suggesting that there is no line broadening in the H_2_O peak due to chemical exchange, and that the same correlation time applies to both resonances. Furthermore, despite the decrease in intensity of the H-3_OH_ resonance, it remains sharp and consistently exhibits coupling behavior to H-3, indicating that it is in a slow exchange process with D_2_O, with complete exchange of the H-3_OH_ occurring over the course of 3 h. 

**Figure 5:**
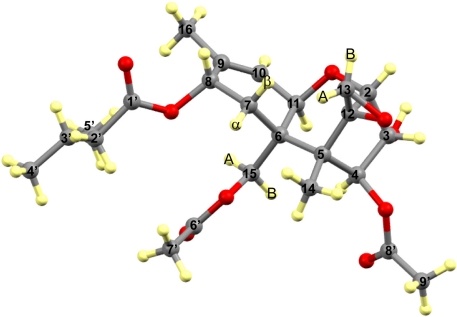
Relative chemical structure of T-2 toxin indicating all ^1^H resonances labelled to show appropriate stereochemistry.

The 1D ^1^H NMR data is consistent with and complements previous NMR findings on T-2 toxin [42,43,44,45]. In the current experiment, the chemical shift and coupling constants of the hydrogens attached directly to the multiple ring skeleton confirms the previously defined stereochemistry. Additionally, NOESY also made it possible to distinguish between H-13_A_ and H-13_B_, whose geminal coupling indicates severe ring strain, as to be expected, for the three-membered epoxide ring. The vicinal couplings of H-7_α_ and H-7_β_ with H-8 confirm that H-7_β_ is gauche to H-8, and that H-7_α_ is perpendicular to H-8, confirming the assignments made by Savard and Blackwell [42,43,44,45]. Long range four- and five-bond coupling constants also imply significant conformational rigidity in solution, especially true between H-7_α_ and H-11. Those long-range couplings to H-10 are primarily the result of efficient π-electron spin propagation due to the nearby double bond. Finally, note that the vicinal couplings (ranging from 6.5-7.5 Hz) in the isovalerate rings are consistent with a low barrier to rotation about the C-C bonds within the isovalerate, which is further supported by the near chemical shift equivalence of H-2′_A_ and H-2′_B_. The complete assignment for T-2 toxin is depicted in Figure 5.

### 3.2. Carbon Spectral Analysis

**Figure 6:**
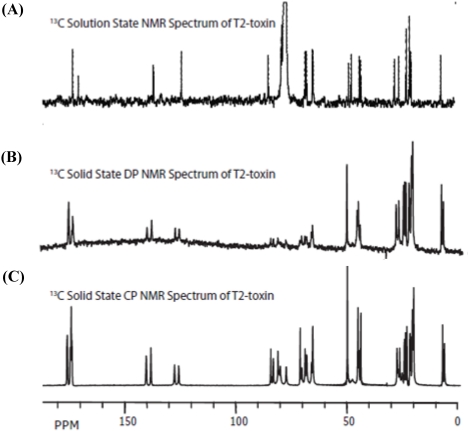
^13^C spectra of T-2 toxin (A) Solution-state spectrum at 75 MHz in CDCl_3_; (B) Direct-polarization (DP) in the solid-state at 125 MHz and MAS of 25 kHz; (C) Cross-polarization (CP) from ^1^H-^13^C (^13^C{^1^H}) at 125 MHz and MAS of 25 kHz.

All of the one-dimensional ^13^C spectra of T-2 toxin are presented in Figure 6. The solution ^13^C-spectrum (Figure 5A) was assigned on the basis of chemical shifts and multiplicities determined by a series of distortionless enhancement by polarization transfer (DEPT) experiments (Data not shown), performed using 45°, 90°, and 135° observation pulses as described by Doddrell *et al.* [46]. The heteronuclear single quantum coherence (HSQC) spectrum confirms both the proton and carbon assignments. Furthermore, the assignment of carbons without attached protons was confirmed by the heteronuclear multiple-bond correlation (HMBC) spectra. The carbon assignment and the cross-peaks of note in the HSQC and HMBC spectra are summarized in Table S5. The heteronuclear correlation methods allow for the unambiguous assignments of all the carbonyl resonances, including those of the carbonyl and methyl carbons of the acetyl side chains, which confirm the assignments previously identified by Savard and Blackwell [43].

**Figure 7:**
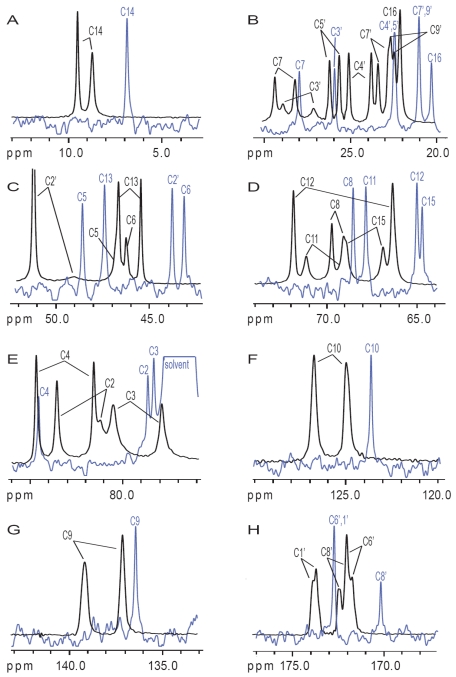
Superposition of the solid-state (black) and solution-state (blue) spectra of the (a) methyl carbons; (b-c) methylene carbons; (d-g) methine, methene, and quaternary carbons; (h) carbonyl carbons, respectively.

The solid-state magic angle spinning (MAS) spectra, obtained by direct polarization (DP; Figure 6B) and cross polarization (CP; Figure 6C), were collected in approximately the same experimental time as the solution spectrum. While the signal-to-noise ratio (S/N) of the DP spectrum is comparable to that of the solution-state spectrum, the S/N is much higher in the CP spectrum compared to either the solution or the DP spectra. It is remarkable that, although the resolution in the solution-state spectrum is expectedly better, the differences between the solid- and solution-state line-widths are as small as 4 Hz (as can observed in Figure 7). This exceptional resolution in the solid-state spectra was made possible by the fast spinning (22 kHz), the high power multiple pulse decoupling, as well as the microcrystallinity of the sample. Under less favorable conditions, line-widths of hundreds of Hz are not unusual. The most significant observation in solid-state, when compared with the solution data, is that all the carbon resonances are twinned. This is consistent with the findings of the X-ray study of T-2 toxin by Gilardi *et al.* [47], where they observed two molecules in the unit cell that differ primarily in the confirmation of the isovalerate side-chain. 

No distinction can be made at this stage between which signals belong to which molecule, as no carbon-carbon correlation information is available, due to the low sensitivity of the natural abundance carbon signal, as well as the long T_1_ relaxation of carbon. A complete assignment will be possible once carbon chemical shift calculations using the Gauge Invariant Plane Augmented Wave (GIPAW) methodology [48], currently being implemented in our group using the ABINIT modeling environment [49], are combined with the carbon-carbon correlations provided by the time demanding ^13^C INADEQUATE [50,51] experiments at natural abundance (which will be the subject of a future contribution).

**Figure 8:**
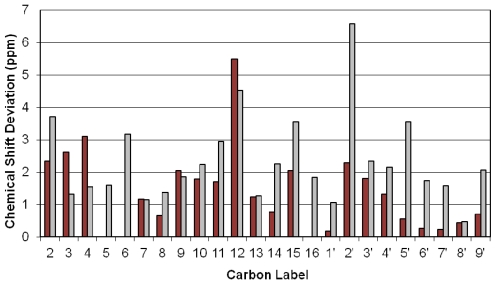
Carbon chemical shift differences in the solid-state (burgundy) between the two conformations in the unit cell, and the average chemical shift difference between solution and solid-state (grey).

At this stage some very interesting observations can be made when comparing solution to solid carbon chemical shifts, as shown in Figure 8. There is no simple correspondence between the carbon chemical shifts of solution to those of the solids (*i.e.*, no fixed difference in frequency), which suggests that they have significant differences in their conformations and in their immediate chemical environment. These differences in the carbon chemical shifts were reconciled through the analysis of close contacts observed in the X-ray structure from Gilardi *et al.* (Figure S3; Table S6) [47].

**Figure 9:**
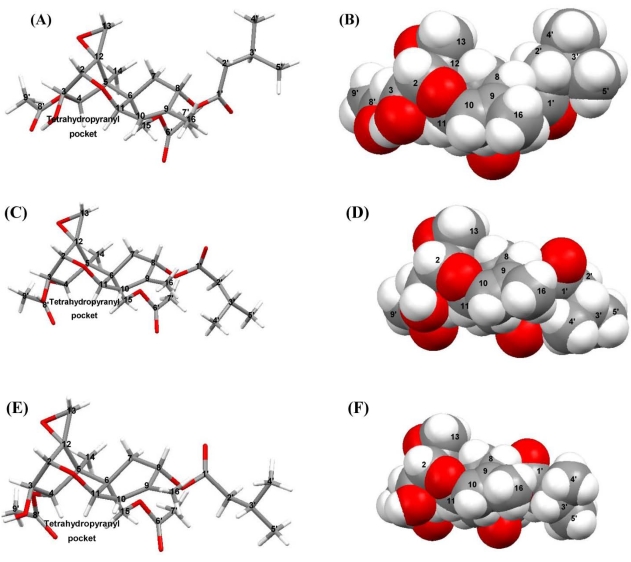
Solution and solid-state conformations. A model of the solution-state structure for T-2 toxin, showing the lowest energy conformation in the: (A) stick model; and (B) van der Waals representation. However, it is important to note here, that the side chains are flexible in the solution structure and are continuously moving, but the core ring structure is rigid; (C) Stick and (D) van der Waals representation for the Solid-state conformation 1, respectively; (E) Stick and (F) van der Waals representation for solid-state conformation 2. Note that the solid-state structure differ only in the torsion angles of the side chains; whereas, the solution state core structure differs substantially from the solid-state structures, particularly in the cyclohexene ring placement.

## 4. Discussion

### 4.1. Hydrogen-Bonding in Solution

Combining the results of the deuterium exchange experiment and the NOESY experiment, some interesting observations arise in regards to the hydrogen-bonding behavior of T-2 toxin. For deuterium exchange experiments, as D_2_O is added to the sample, it rapidly interchanges with free water in order to form HDO. For fast exchange between H_2_O and HDO one would expect to observe a single broad line, due to the coupling of the HDO proton to the deuterium nucleus which confers a fine triplet structure to the peak, and shifts the resonance slightly upfield. However, our observations indicate that the conversion from H_2_O to HDO is on a much slower timescale, given by the rather distinct and sharp lines observed for both H_2_O and HDO (Figure 2B). The coexistence of these peaks, suggests that at least a portion of the water in the sample is in a bound configuration, preventing free exchange with D_2_O. Furthermore, the linewidth for the H-3_OH_ resonance remains consistent over the course of the exchange experiment, were H-3_OH_ to be undergoing fast exchange with water, a broad featureless line would be expected. The sharpness of the H-3_OH_ peak, as well as the coupling observed between this proton and H-3, signify that H-3_OH_ is most likely involved in a hydrogen bonding interaction of some kind. This data suggest that the H_2_O is bound to T-2 toxin; thereby, preventing the water and H-3_OH_ from exchanging rapidly with D_2_O.

**Figure 10:**
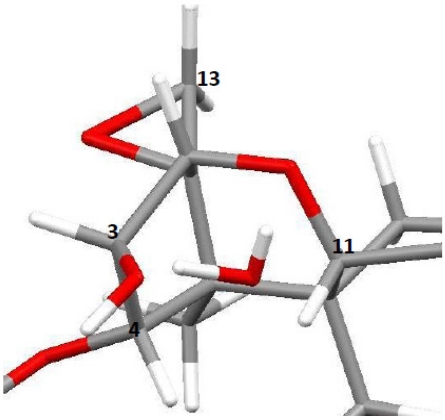
Proposed placement of water in the tetrahydropyranyl pocket of T-2 toxin. Cross-relaxation was observed between water and H-3, H-4 and H-11, indicating close proximity; chemical exchange between water and H-3_OH_ was also observed.

The orientation of the bound water, with respect to the molecule, can be determined through analysis of the NOESY spectrum. It is important to note that in spectra where chemical exchange is a factor, second-order artifacts known as saturation transfer peaks, cannot be ruled out; therefore, it is necessary to analyze any crosspeaks to water with care in order to determine whether they are true first-order cross-relaxation signals, or artifacts due to saturation transfer. Saturation transfer peaks for small molecules, such as T-2 toxin, have the same phase as the diagonal autocorrelation peaks; whereas, the longer correlation times of large macromolecules, such as proteins and nucleic acids, result in saturation transfer peaks that have the same phase as conventional NOE peaks [52]. Therefore, it is expected that any saturation transfer peaks in T-2 toxin would appear negative due to the short correlation time of the molecule. Additionally, saturation transfer will not occur if the rate of chemical exchange is on the same order or longer than the rate of longitudinal (T_1_) relaxation [53]. Considering that the rate of chemical exchange, as observed in the deuterium experiment described above, is estimated to be relatively long, the observation of saturation transfer peaks for this system is highly unlikely. The observed crosspeaks to water for H-3, H-4, and H-11 are all positive (Figure 4), and consequently must arise due to cross-relaxation. This suggests that H-3, H-4, and H-11 are all in close proximity to water. Thus, at least one water molecule is bound to T-2 toxin, and lies within the tetrahydropyranyl pocket located on the opposite side of the molecule which respect to the epoxide (Figure 10).

### 4.2. Solid-State Configurations

The two contributions to the solid-state NMR spectrum were compared to the two molecules in the unit cell of the X-ray crystallography structure (Figure S3). Even though H-bonds cannot be observed directly by X-ray methods, close contacts can be used to infer where they may occur (Table S6). Accordingly, there are several hydrogen bond interactions inferred between the two conformations in the unit cell. The shortest contact occurs between the H-3_OH_ of conformation 2 and O-12 (the epoxide oxygen) of conformation 1 (top H-bond). This interaction would dramatically affect the chemical shifts of C-3 in conformation 2, and of C-12 and C-13 in conformation 1. Other significant short contacts occur between the hydroxyl hydrogen (H-3_OH_) of conformation 1 and two of the oxygens of conformation 2, namely the ring oxygen (O-1) and the hydroxyl oxygen (O-3) (bottom H-bond). This interaction would affect the chemical shifts of C-2, C-3, C-4, C-11, and C-12. The chemical shift differences in the solid-state carbon spectra are consistent with these predictions. Since C-12 is in close proximity to both interactions, its chemical shift appears to be the most affected, followed by those of C-2, C-3, C-4 and C-11 (Figure 8). Additionally, the significant differences in the chemical shifts of C-10 and C-11 cannot be ignored, and are likely a secondary influence from the rather large effect seen on C-12, as there is significant π-electron density nearby to transmit such effects. 

Short contacts resulting in van der Waal type interactions also occur between H_5’_ and H_16_ as well as H-2′_A_ and H-5′ on conformations 1 and 2, respectively; Table S6. One would expect the chemical shift of C-5′, C-2′ and C-16 to be affected accordingly; however, only the chemical shifts of C-2′, and unexpectedly, C-9, are affected by this interaction, while C-5′ and C-16 are not. Finally, significant contact involving non-hydrogen bonding dipolar interactions between H-13_B_ and H-15_A_ as well as H-14 and H-3_OH_ on conformations 1 and 2, respectively, should also be considered (Table S6). As a consequence the chemical shifts of C-13, C-14 and C-15 should be altered; however, it seems only C-15 is affected. 

The exchange crosspeak between water and the H-3_OH_ resonance of T-2 toxin in the solution ^1^H NOESY spectrum strongly supports the bottom H-bonding interaction but not the top H-bond (Figure 4). The difference between the carbon chemical shifts in solution with the average shifts in the solid-state agree with this observation, this is particularly striking for C-12. The rather large differences in carbon chemical shifts seen for the isovalerate and acetyl side-chains resonances is consistent with their high conformational flexibility in the solution state, while in the solid-state they are locked into only two conformations corresponding to molecules 1 and 2, as defined in the X-ray structure, which is indicated by the distinct chemical shifts observed in the solid-state. 

### 4.3. Ribosomal Interaction

The conformational differences observed for T-2 toxin in solution and solid-state, as well as the hydrogen bonding behavior for this compound provides some curious insights into the biological behavior of T-2 and similar trichothecenes. Of particular interest is the interaction with the eukaryotic ribosome.

Inhibition of protein synthesis by trichothecenes occurs via interactions of the toxin with the peptidyl transferase center (PTC) of the eukaryotic ribosome, similar to the mode of action of many antibiotics with the prokaryotic ribosome. Trichothecenes have been shown to make contacts with multiple proteins in the PTC, the most important of which appears to be the contacts with RPL3 [19]. Analysis of a resistant version of RPL3 suggests that the inhibition most likely occurs through an interaction of the trichothecene with the tryptophan residue (W255 in *Saccharomyces cerevisiae*) located on the W-finger [21]. It is important to note, that this hypothesis is based on mutational studies in yeast, whereby a mutation to W255C or W255R confers resistance. It is curious that a ribosomal point mutation confers resistance to the toxins, suggesting that many of the other toxicological effects observed may in fact be downstream consequences of the inhibition of protein synthesis resulting from the inability to replenish the cellular machinery required to maintain these important processes.

Extensive studies have been conducted which reveal that the epoxide ring is required for toxicity and is believed to form the primary interaction in the PTC center in order to disrupt protein synthesis [29,54]. Opening of the ring leads to a complete loss of toxic activity [29,55]. The epoxide ring remains stable to nucleophilic attack in solution, due to the obstruction of carbon C-12 on the side opposite from the epoxide oxygen [29,54,55,56]. The 3-dimensional solution-state structure depicted in Figure 9 clearly demonstrates that the epoxide ring of T-2 toxin is completely obscured on the side where the nucleophilic attack by an S_N_2 like mechanism would have to occur. This is further emphasized by the van der Waals representation of the molecule, where it becomes clear that the presence of a nucleophilic ion, or a bridging water molecule (as seen in the solid-state structure), is stereochemically mired, despite the rigidity conferred by the water bridge in the tetrahydropyranyl ring. This effect is not observed in the solid-state conformation, due to the more relaxed conformation in the cyclohexene ring. This relaxed solid configuration, allows for the presence of a water molecule to sit in the “epoxide pocket”, or top of the molecule. An interaction with water at this location on the molecule may serve as a bridge between the epoxide and the W-finger of RPL3. 

The X-ray structure was used to infer the hydrogen bonding interactions occurring in the solid state, based on close contacts [47]. These interactions have profound effects on the chemical shifts of the nearby carbons. The solid-state carbon chemical shifts given here, presented for the first time, are consistent with the X-ray structure predictions, thus supporting the existence of the bottom hydrogen bonding interaction, as well as a hydrogen bonding interaction with the epoxide function (top). The comparison of the chemical shifts in solution- and solid-state and the lack of NOESY evidence for the top hydrogen bonding interaction, indicate that the hydrogen bonding in solution differs substantially from that in the solid-state. Furthermore, these conformational differences, observed in T-2 toxin in the solid-state, suggest that the epoxide ring is now available to hydrogen bond with another molecule. The carbon chemical shifts in both conformations in the solid state of C-12, the proposed target of nucleophilic attack, are shifted significantly downfield, suggesting that they are further deshielded and that the hydrogen bonding at the top of the molecule could potentially be an interaction with a water molecule.

These findings are important when considering the mechanistic descriptions of the biological interaction of trichothecenes. In particular, hydrogen bonding interactions become important when considering binding affinities and bond geometries of protein-ligand complexes. The interaction of trichothecenes with the ribosome creates a particularly interesting scenario, as the different substituent groups off the core trichothecene structure have been shown to inhibit the different stages of protein synthesis. It is believed that all trichothecenes interact with the ribosomal protein L3 (RPL3) [19]; however, the different mechanisms of ribosomal inhibition are most likely linked to the different types of hydrogen bonding interactions that the substituent groups are able to form with the other surrounding atoms in the active site.

Another important factor to consider is the functionality of the ribosome, and the availability of water molecules throughout the different stages of protein synthesis. The ratcheting behavior of the ribosome works to reorganize water within the PTC pocket, and shield peptide bond formation from bulk water [57]. Furthermore, the removal of water lowers the entropy of the system; thus providing a dramatic increase in free energy to counter the enthalpically unfavorable peptide bond reaction. It is also important to note, that the change in hydration within the PTC pocket could also result in a change in the pH of the environment within the pocket. With the new information regarding the different conformations of T-2 toxin in solution versus the solid-state, the water environment within the PTC must be taken into account, as the change in hydration of the system may help to modulate a conformational change in T-2 toxin from the solution-state structure to a solid-like structure where the epoxide is free to hydrogen bond. Trichothecenes have been shown to exhibit different structural configuration based on the solvent to which they are exposed [31]. A similar situation also occurs with the antibiotic virginiamycin M1, which also binds to the ribosome, and exhibits different structural configurations depending primarily on the hydrophobicity of the solvent system [32,33]. Furthermore, the change in free energy of the system may serve to drive the mechanism of inhibition. 

RPL3 has a high degree of sequence and structure conservation across all three domains of life. The W-finger, in particular, is universally conserved, and the tryptophan is in close proximity to the active site of the PTC, where peptide bond formation occurs [58,59]. Individual mutations to the amino acids of the W-finger seem to indicate that it functions as a sensor for A-site occupancy and helps to synchronize aa-tRNA accommodation, transfer, and translocation [20]. Meskaukas and Dinman proposed that when no aa-tRNA is present, the W-finger extends into the A-site, and when the correct aa-tRNA is introduced, the W-finger is displaced [20]. It is important to note that the *N*-terminal extension only appears to be present among eukaryotic and archaeal species [19], which may explain why the majority of prokaryotic species are resistant to trichothecene toxicity.

To the best of our knowledge, no kinetic studies have been performed to confirm the interaction of trichothecenes with RPL3. It would be beneficial to study the interaction of trichothecenes with free L3 to determine whether interaction is possible in solution; however, since it is well known that the epoxide ring of trichothecenes is both heat and pH stable in the solution state, it is difficult to estimate exactly how such a reaction would take place [60]. It is our belief that the solid-state trichothecene conformation is required for the interaction to have toxic effects on the system. 

**Figure 11:**
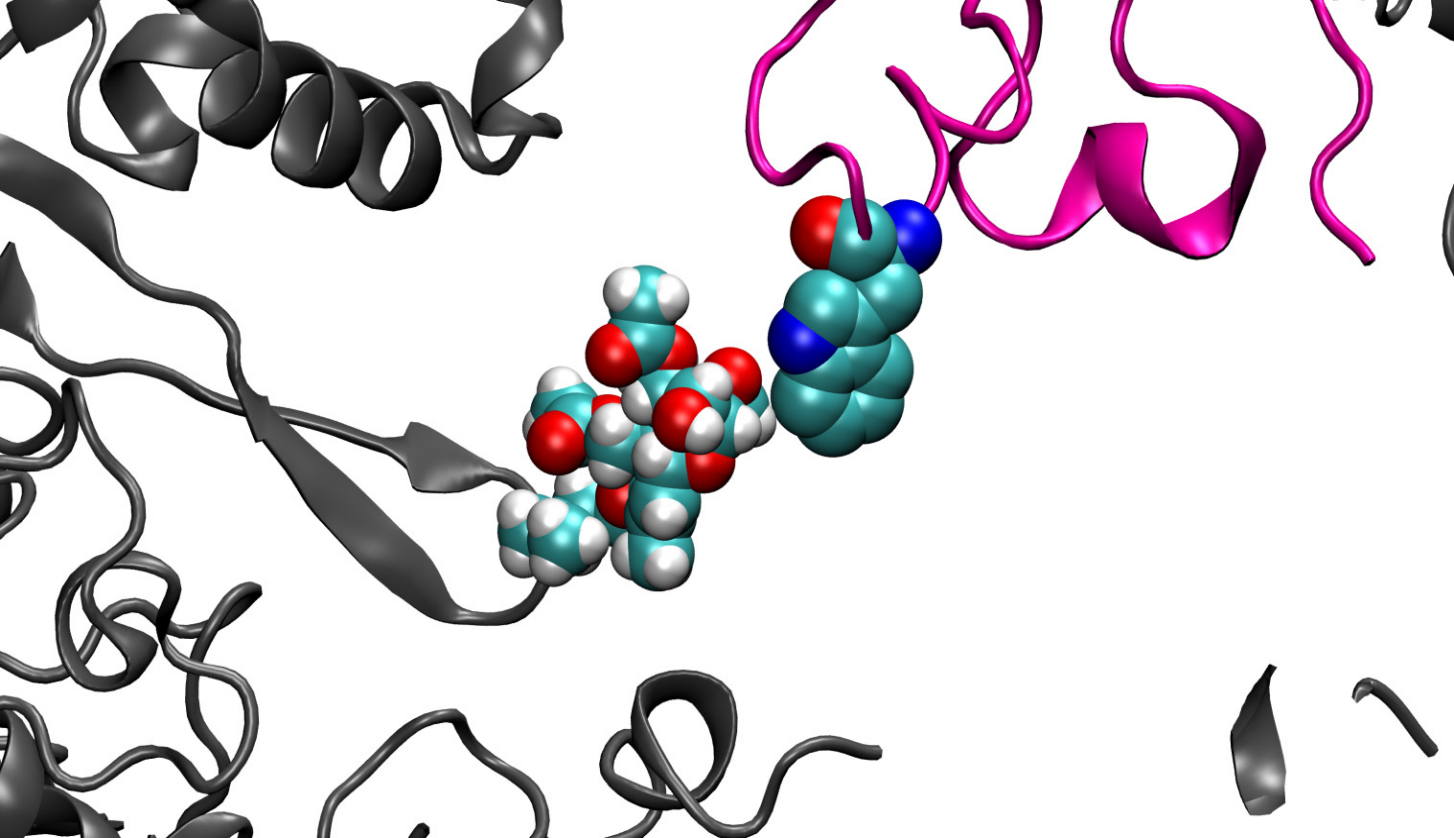
Model representing the proposed transient interaction of the trichothecene toxin with W255 of the W-finger of ribosomal protein, RPL3. RPL3 is shown in magenta, with the tryptophan residue, W255, shown explicitly. The other proteins of the PTC which are nearby are depicted in grey. The van der Waals representation was used to demonstrate the close contacts of the epoxide with the nitrogen of the tryptophan.

It is our hypothesis that the initial interaction of the trichothecene with the ribosome occurs in solution. At this stage a transient association of the trichothecene with W255, in *S. cerevisiae*, at the W-finger domain of RPL3 occurs (Figure 11). Upon peptide bond formation, the ribosome sequesters water from the PTC pocket, changing the level of hydration. The change in hydration results in a conformational change in the trichothecene structure, allowing for a water or salt bridge to form between the epoxide ring of the toxin and the tryptophan residue of the W-finger. Once the initial interaction of the trichothecene occurs with RPL3, the substituent groups off the core trichothecene structure interact with the surrounding nucleic acids and amino acid residues to block either the A-, P- or E-site and inhibit initiation, elongation or termination respectively.

## 5. Conclusions

A complete proton and carbon NMR analysis was performed to verify the stereochemistry of T-2 toxin, and to confirm the relative assignment of all the methylene protons, acetyl, methyl and carbonyl resonances. Furthermore, full simulation of the proton spectrum provided all the spectral parameters to within 0.02-0.06 Hz accuracy, and led to useful structural and dynamic insights such as: the rigidity of the ring structure, the conformational flexibility of the side chain, and the ring strain in the epoxide group. Most notably, exchange dynamics were observed between water and H-3_OH_ via EXSY cross peaks, lending support to existence of a hydrogen bonding interaction between T-2 toxin and water. Additionally, cross-relaxation peaks in the NOESY spectrum observed between water and H-3, H-4 and H-11 indicate that the water is bound within the tetrahydropyranyl pocket present on the side of the ring opposite to the epoxide functionality. Deuterium exchange experiments were performed to address whether water was bound to the toxin or free in solution. Sharp coupled peaks and slow chemical exchange between H_2_O and D_2_O indicate that there is at least one water molecule bound to T-2 toxin through a hydrogen bond with the hydroxyl hydrogen on C-3, which is also observed to be in the slow chemical exchange regime. This hydrogen bond is likely involved in a water-bridging interaction with the molecule and functions to stabilize and rigidify the trichothecene core.

A solid-state NMR analysis of the T-2 toxin structure was performed, which not only confirms the presence of two distinct polymorphic contributions to T-2 toxin, as originally observed in the X-ray crystallographic analysis performed by Gilardi [47]; but also indicates that an interaction with a water molecule may also be occurring in the solid-state. It is also important to note that both solid-state configurations of T-2 toxin differ from that of the solution-state structure, as interpreted from the differences in the chemical shift of the ^13^C resonances. 

With these new insights into the three-dimensional structure of T-2 toxin in both the solution- and solid-state, it is possible to begin piecing together the mechanism for toxicity of trichothecene toxins, starting with the ribosome. As such, we have proposed a novel mechanism for the interaction of T-2 toxin with the eukaryotic ribosome, which ideally, will be applicable to other toxins of this family. Undoubtedly, this new information is a stepping stone towards the guided development of trichothecene resistance genes which can be developed for the introduction into transgenic plants, such as those previously detected by groups such as Mitterbauer *et al.* for the engineering of Triticale cultivars [61]. The development of trichothecene resistance genes is essential to the prevention of crop loss due to Fusarium Head Blight and root rot, and will help to circumvent the occurrence of alimentary toxic aleukia, and other related ailments. 

## Supporting Information Available

Supplementary Data include Supplemental Experimental Details, Tables of ^1^H and ^13^C Chemical Shifts and Coupling Constants. This information is available free of charge via the Internet at http://pubs.acs.org.

## Supplementary Files

Supplementary File 1: PDF-Document (PDF, 756 KB)
