# Supplementary material for: Hydrogen-Bonding Interactions in T-2 Toxin Studied Using Solution and Solid-State NMR
Source: Toxins (Basel). 2011 Oct 21;3(10):1310–31. doi: 10.3390/toxins3101310 (PMC3210463; doi:10.3390/toxins3101310)
Supplement: Supplementary File 1: — PDF-Document (PDF, 756 KB) [file toxins-03-01310-s001.pdf]

Supplementary Information

Praveen Chaudhary [1,†], Roxanne A. Shank [1,2,†], Tony Montina [1], James T. Goettel [1], Nora A. Foroud [2], Paul Hazendonk [1,*] and François Eudes [2,*]

[1] Department of Chemistry and Biochemistry, University of Lethbridge, 4401 University Drive West, Lethbridge AB T1K 3M4, Canada; E-Mails: praveen.chaudhary@uleth.ca (P.C.); shankr2@uleth.ca (R.A.S.); tony.montina@uleth.ca (T.M.); james.goettel@uleth.ca (J.T.G.);

[2] Lethbridge Research Centre, Agriculture and Agri-Food Canada, 5403-1st Avenue South, Lethbridge AB T1J 4B1, Canada; E-Mail: nora.foroud@agr.gc.ca

[†] These authors contributed equally to the work.

* Authors to whom correspondence should be addressed; E-Mails: paul.hazendonk@uleth.ca (P.H.); francois.eudes@agr.gc.ca (F.E.); Tel.: +1-403-329-2657 (P.H.); +1-403-317-3338 (F.E.); Fax: +1-403-329-2057 (P.H.); +1-403-382-3156 (F.E.).

Received: 19 August 2011; in revised form: 28 September 2011 / Accepted: 11 October 2011 / Published: 21 October 2011

Abstract: The structure of T-2 toxin in the solid-state is limited to X-ray crystallographic studies, which lack sufficient resolution to provide direct evidence for hydrogen-bonding interactions. Furthermore, its solution-structure, despite extensive Nuclear Magnetic Resonance (NMR) studies, has provided little insight into its hydrogen-bonding behavior, thus far. Hydrogen-bonding interactions are often an important part of biological activity. In order to study these interactions, the structure of T-2 toxin was compared in both the solution- and solid-state using NMR Spectroscopy. It was determined that the solution- and solid-state structure differ dramatically, as indicated by differences in their carbon chemical shifts, these observations are further supported by solution proton spectral parameters and exchange behavior. The slow chemical exchange process and cross-relaxation dynamics with water observed between the hydroxyl hydrogen on C-3 and water supports the existence of a preferential hydrogen bonding interaction on the opposite side of the molecule from the epoxide ring, which is known to be essential for trichothecene toxicity. This result implies that these hydrogen-bonding interactions could play an important role in the biological function of T-2 toxin and posits towards a possible interaction for the trichothecene class of toxins and the ribosome. These findings clearly illustrate the importance of utilizing solid-state NMR for the study of biological compounds, and suggest that a more detailed study of this whole class of toxins, namely trichothecenes, should be pursued using this methodology.

Keywords: T-2 toxin; trichothecene; NMR; hydrogen-bonding; ribosome; toxin; epoxide; water bridging; deuterium exchange; chemical exchange

Table S1. ^{1}H Chemical Shifts of T-2 Toxin in $CDCl_3$.

Label	Chemical Shift (ppm)	Frequency (Hz)	J partners (Spectral Analysis)	COSY	NOESY
2	3.681 (10^{-5}) [1]	1104.417 (0.003) [1]	3, 3_{OH} 14	3,3_{OH},14	3,13_B
3	4.160 (10^{-5})	1248.079 (0.003)	2,3_{OH},4,13_A, 14	2,3_{OH},4	2,3_{OH},4,H_2O
3_{OH}	3.100 (10^{-5})	930.117 (0.003)	2,3,4	2,3	3,4,11,H_2O
4	5.348 (10^{-5})	1604.452 (0.003)	3,3_{OH},14,13_B	3,9′,13_B,14	3,3_{OH},11,14, 15_{AB},9′,H_2O
7_α	1.886 [2] (10^{-5})	565.959 (0.003)	7_β,8,11	7_β,8,11	7_β,8,14
7_β	2.408 [2] (10^{-5})	722.688 (0.003)	7_α,8, 15_B	7_α,8,15_B	7_α,8,13_A
8	5.298 (10^{-5})	1591.419 (0.003)	$7_{\alpha\beta}$,10,16	$7_{\alpha\beta}$,10,16	$7_{\alpha\beta}$,16,2′
10	5.818 (10^{-5})	1741.845 (0.003)	8,11,16	8,11,16	11,16
11	4.355 (10^{-5})	1310.316 (0.003)	7_α,10,15_B,16	7_α,10,16	3_{OH},4,10,H_2O
13_A	2.809 [2] (10^{-5})	837.234 (0.003)	3,13_B	13_B,14	7_β,13_B,14
13_B	3.046 [2] (10^{-5})	914.074 (0.003)	4,13_A	4,13_A,14	2,13_A
14	0.817 (10^{-5})	242.706 (0.002)	2,4	2,4,13_{AB},15_A	4,7_α,13_A,15_A
15_A	4.293 [2] (10^{-5})	1220.756 (0.003)	15_B	14,15_B,7′	4,15_B
15_B	4.066 [2] (10^{-5})	1292.338 (0.003)	15_A,7_β,11	7_β,7′,15_A	4,15_A
16	1.748 (10^{-5})	524.420 (0.002)	8,10,11	8,10,11	8,10

[1] Standard Deviation from spectral analysis using Spin Works [41].
[2] Labelling convention as taken from Savard and Blackwell, Can.J.Chem (1987) [42].

Table S2. ^{1}H Coupling Constants of T-2 Toxin in $CDCl_3$ determined through simulations performed with SpinWorks 3.1.6.

Label	Coupling (Hz)	Comment/Assignment
$^3J_{2,3}$	4.946 (0.005) [1]	2 is gauche to 3
$^4J_{2,3OH}$	−0.294 (0.005)	
$^4J_{2,14}$	−0.304 (0.004)	
$^3J_{3,3OH}$	2.909 (0.005)	3 is gauche to 3-OH
$^3J_{3,4}$	2.885 (0.005)	3 is gauche to 4
$^5J_{3,13A}$	0.211 (0.005)	
$^5J_{4,13B}$	0.160 (0.005)	
$^4J_{3OH,4}$	−0.293 (0.005)	

$^{4}J_{4,14}$	−0.277 (0.003)	
$^{2}J_{7\alpha,7\beta}$	−15.015 (0.006)	Geminal coupling typical of sp^3 carbon
$^{3}J_{7\alpha,8}$	1.248 (0.006)	7_α nearly perpendicular to 8
$^{4}J_{7\alpha,11}$	−1.598 (0.006)	W configuration
$^{3}J_{7\beta,8}$	5.718 (0.006)	7_β is gauche to 8
$^{4}J_{7\beta,15B}$	−0.446 (0.006)	W configuration in one rotamer
$^{4}J_{8,10}$	−0.979 (0.006)	Typical of H on sp^2 carbon
$^{4}J_{8,16}$	−0.700 (0.003)	Indicative of rigidity of ring
$^{3}J_{10,11}$	5.892 (0.006)	10 is gauche to 11
$^{4}J_{10,16}$	−1.451 (0.003)	Typical of H and CH_3 on sp^2 carbon
$^{4}J_{11,15B}$	−0.588 (0.006)	W configuration in one rotamer
$^{5}J_{11,16}$	0.739 (0.003)	Typical of CH_3 on sp^2 carbon
$^{2}J_{13A,13B}$	3.971 (0.005)	Geminal coupling indicating ring strain
$^{2}J_{15A,15B}$	−12.835 (0.006)	Geminal coupling typical of sp^3 carbon

[1] Standard Deviation from spectral analysis using Spin Works [41].

Table S3. ^{1}H Chemical Shifts of Side-Chain Groups on T-2 Toxin in $CDCl_3$.

**Label**	**Chemical Shift (ppm)**	**Frequency (Hz)**
$2'_A$	2.154 (0.0002) [1]	646.21 (0.06) [1]
$2'_B$	2.159 (0.0002)	647.78 (0.06)
3′	2.107 (0.0003)	631.99 (0.08)
4′	0.970 (0.0002)	290.97 (0.05)
5′	0.961 (0.0002)	288.38 (0.05)
7′	2.041 (0.0001)	612.30 (0.02)
9′	2.151 (0.0001)	645.22 (0.02)

[1] Standard Deviation from spectral analysis using Spin Works [41].

Table S4. ^{1}H Coupling Constants of Side-Chain Groups on T-2 Toxin in $CDCl_3$

**Label**	**Coupling (Hz)**	**Comment/Assignment**
$^{2}J_{2'A,2'B}$	-14.03 (0.10) [1]	Typical geminal coupling of sp^3 carbon
$^{3}J_{2'A,3'}$	7.33 (0.10)	Rotationally averaged
$^{3}J_{2'B,3'}$	7.56 (0.10)	Rotationally averaged
$^{3}J_{3',4'}$	6.77 (0.10)	Rotationally averaged
$^{3}J_{3',5'}$	6.61 (0.10)	Rotationally averaged

[1] Standard Deviation from spectral analysis using Spin Works [41].

Figure S1. ^{13}C to ^{1}H HSQC in $CDCl_3$.

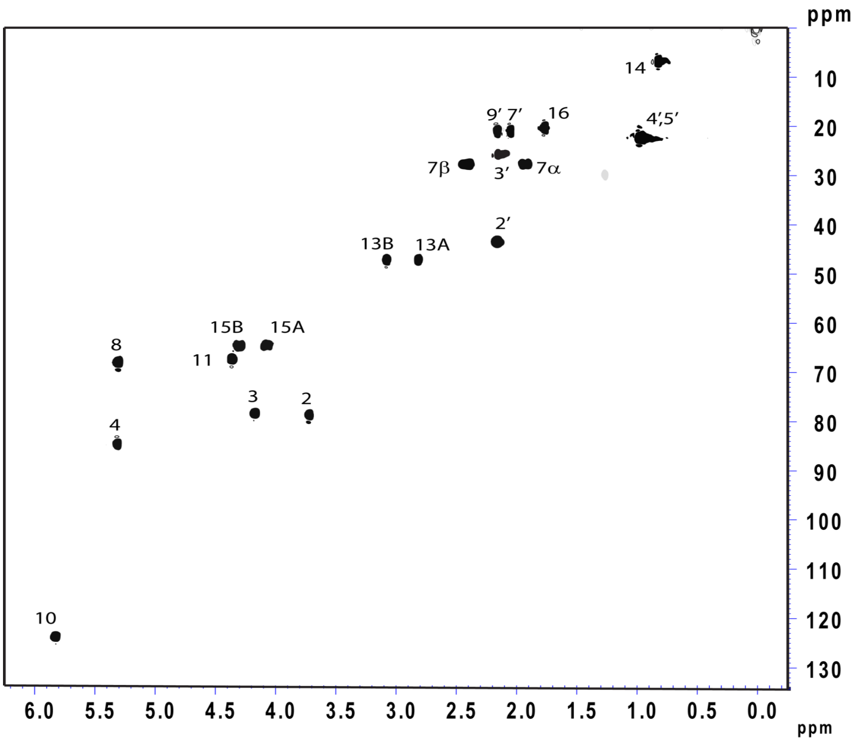

Figure S2. ^{13}C to ^{1}H HMBC in $CDCl_3$.

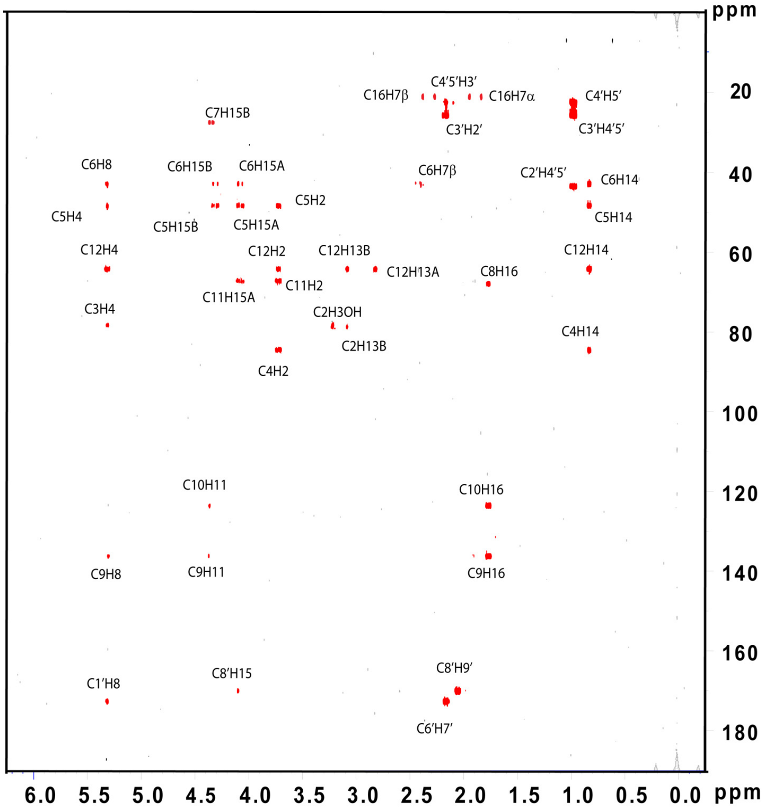

Table S5. ^{13}C Chemical Shifts.

Label	Solution (ppm)	HSQC	HMBC	Solid 1 (ppm)	Solid 2 (ppm)
2	78.72	2	3_{OH},13_{B}	81.25	83.59
3	78.40	3	4	77.94	80.57
4	84.60	4	2,14	81.6	84.7
5	48.39		2,4,14,15_{AB}	46.8	46.8
6	42.94		7_{β},8,14,15_{AB}	46.1	46.1
7	27.75	$7_{\alpha\beta}$	15_{B}	28.32	29.49
8	68.02	8	16	69.07	69.73
9	136.32		8,11,16	137.15	139.2
10	123.70	10	11,16	125.03	126.82
11	67.34	11	2,15_{A}	69.45	71.15
12	64.59		2,4,13_{AB},14	66.36	71.86
13	47.21	13_{AB}		45.33	46.56
14	6.88	14		9.51	8.73
15	64.31	15_{AB}		66.85	68.9
16	20.35	16	$7_{\alpha\beta}$	22.19	22.19
1′	172.73		8	173.71	173.9
2′	43.58	2′	4′,5′	49.00	51.30
3′	25.78	3′	2′,4′,5′	27.22	29.02
4'	22.37	4′	3′,5′	23.87	25.18
5′	22.45	5′	3′	25.73	26.29
6′	170.13		7′	171.74	172
7′	21.07	7′		22.53	22.76
8′	172.70		15, 9′	172	172.44
9′	21.04	9′		22.76	23.47

Figure S3. Crystal Structure of T-2 Toxin of conformations 1 and 2 in the unit cell. Top view (top) and Bottom view (bottom).

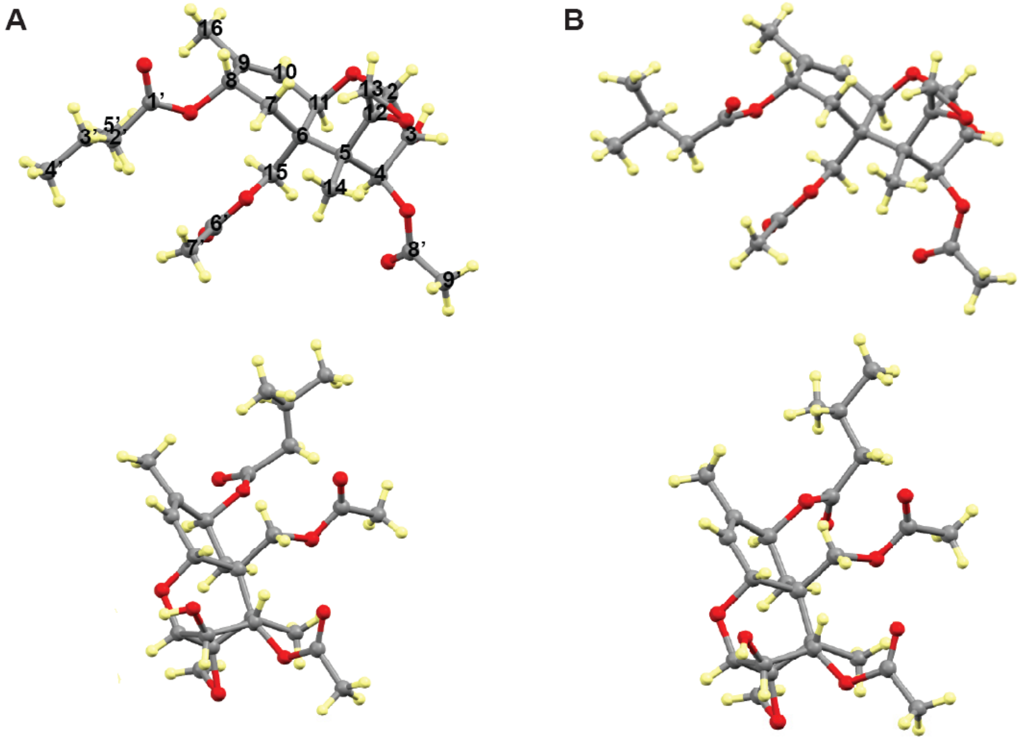

Table S6. Close contact in T-2 Toxin X-ray Structure.

ATOM1	ATOM2	Length	Carbons	Carbons	Type
O-12(1)	H-3$_{OH}$(2)	1.88	C-12,C-13	C-3	H-bond
H-3$_{OH}$(1)	O-1(2)	2.203	C-3	C-2,C-11	H-bond
H-3$_{OH}$(1)	O-3(2)	2.299	C-3	C-3	H-bond
H-16(1)	H-5′(2)	2.32	C-16	C-5′	VDW
H-5′(1)	H-2′$_{A}$(2)	2.335	C-5′	C-2′	VDW
H-13$_{B}$(1)	H-15$_{A}$(2)	2.367	C-13	C-15	DP
H-14(1)	H-3$_{OH}$(2)	2.384	C-14	C-3	DP
H-14(1)	H-9′(2)	2.399	C-14	C-8′,C-9′	VDW/DP
O-3(1)	H-8(1)	2.512	C-3	C-1',C-8	DP
H-11(1)	O-1′(1)	2.577	C-11	C-1′,C-2′	DP
O-8′(1)	H-7′(2)	2.588	C-8′,C-9′	C-6′,C-7′	DP
H-7′(1)	O-6′(2)	2.593	C-7′,C-6′	C-6′,C-7′	DP
H-9′(2)	O-8′(2)	2.595	C-9′,C-8′	C-8′,C-9′	DP
H-9′(1)	O-4(2)	2.666	C-9′,C-8′	C-4′,C-8′	DP
H-3(1)	O-3(2)	2.679	C-3	C-3	H-bond
H-13$_{B}$(2)	O-6′(2)	2.704	C-12,C-13	C-6′,C-7′	DP
O-12(1)	O-3(2)	2.737	C-12,C-13	C-3	H-bond

H-3$_{OH}$(1)	C-11(2)	2.754	C-3	C-11	H-bond
C-6′(1)	H-7′(2)	2.76	C6′,C7′	C6′,C7′	DP
C-13(1)	H-3$_{OH}$(2)	2.763	C-12,C-13	C-3	H-bond
C-13(1)	H-15$_{A}$(2)	2.835	C-13	C-15	DP
O-3(1)	O-1(2)	2.89	C-3	C-2,C-11	H-bond
O-3(1)	O-3(2)	2.968	C-3	C-3	H-bond
C-3(1)	O-3(2)	3.159	C3	C3	H-bond
